# The Evolving Role of Bispecific Antibodies in Oncogene-Driven NSCLC

**DOI:** 10.3390/cancers18142197

**Published:** 2026-07-08

**Authors:** Jun Chih Wang, Daniel Rosas, Luis E. Raez

**Affiliations:** Memorial Healthcare System, Pembroke Pines, FL 33026, USAlraez@mhs.net (L.E.R.)

**Keywords:** bispecific antibodies, non-small-cell lung cancer, EGFR, MET, amivantamab, zenocutuzumab, tyrosine kinase inhibitors, targeted therapy, oncogene-driven NSCLC, antibody–drug conjugates

## Abstract

Bispecific antibodies (bsAbs) have emerged as a novel therapeutic class in oncogene-driven non-small-cell lung cancer (NSCLC), designed to simultaneously target multiple signaling pathways and overcome resistance mechanisms associated with tyrosine kinase inhibitors (TKIs). Unlike small-molecule TKIs, bsAbs enable dual receptor blockade and immune effector engagement, offering a mechanistically distinct advantage in the context of tumor heterogeneity and bypass signaling. This review summarizes the structural and biological principles underlying bsAb design, with a focus on clinically approved agents such as amivantamab (EGFR/MET) and zenocutuzumab (HER2/HER3) and a growing pipeline of investigational agents. We evaluate key clinical evidence from Phase I-III trials including CHRYSALIS, PAPILLON, MARIPOSA, MARIPOSA-2 and eNRGy, and compare the efficacy, toxicity, and CNS penetration profile of bsAbs relative to TKIs and antibody–drug conjugates (ADCs). While bsAbs demonstrate meaningful clinical activity, particularly in TKI-resistant disease and molecularly defined subsets such as EGFR exon 20 insertions and NRG1 fusions, their limitations, including intravenous administration, increased immune-mediated and thromboembolic toxicity, currently preclude replacement of TKIs in most settings. Collectively, available evidence supports a complementary role for bsAbs within evolving multimodal treatment paradigms, particularly in combination strategies. Future directions include biomarker-driven patient selection, improved drug engineering and integration into adaptive therapeutic sequencing frameworks.

## Simple Summary

Lung cancer remains the most common cause of cancer death worldwide. In non-small-cell lung cancer, researchers have identified many specific genetic mutations that drive tumor growth. Therapies such as tyrosine kinase inhibitors, which target these mutations, are often effective, but tumors eventually develop resistance to them through a variety of mechanisms. This review examines a novel treatment strategy, bispecific antibodies, which lock onto two targets on cancer cells at the same time and recruit the body’s own immune system to help eradicate the tumor. By reviewing the efficacy and safety data from the latest clinical trials of these antibodies, we synthesize a treatment algorithm that clarifies where they fit within the existing treatment landscape. This overview should help clinicians and researchers choose treatment more precisely and design more rigorous future studies for people with lung cancer.

## 1. Introduction

Lung cancer remains the leading cause of cancer-related mortality worldwide, accounting for approximately 1.8 million deaths annually [1]. Non-small-cell lung cancer (NSCLC) comprises roughly 85% of all lung cancers, with adenocarcinoma representing the predominant histological subtype in which actionable genomic drivers are identified [2]. Over the past two decades, the systematic identification of oncogenic driver alterations has fundamentally transformed the therapeutic landscape into an era of precision oncology [1,3].

The current targetable oncogene landscape in advanced NSCLC encompasses EGFR mutations (18–51%), KRAS G12C (~13% in Western populations), ALK rearrangements (3–7%), MET exon 14 skipping mutations (3–4%), RET rearrangements (1–2%), ROS1 rearrangements (1–2%), HER2 mutations or amplification (1.5–3%), BRAF V600E mutations (~1–3%), and NTRK1/2/3 fusions (<1%) [3,4]. These mutations show distinct clinicopathological associations, are generally mutually exclusive, and each represents a tractable therapeutic target [5]. TKIs directed against these drivers have achieved substantial improvements in progression-free survival (PFS) and quality of life [3,6]. See Table 1(A) for a summary of all major oncogenic drivers, their epidemiology, and approved agents with their registration trials.

Despite these advances, acquired resistance to TKIs is virtually universal [32,33,37]. Resistance mechanisms may be broadly categorized as: (1) on-target: secondary kinase mutations (e.g., EGFR C797S, ROS1 G2032R); (2) off-target: bypass pathway activation via MET/HER2 amplification, HER3 upregulation, or AXL overexpression; (3) downstream signaling: KRAS/BRAF mutations activating the MAPK/PI3K cascades; and (4) phenotypic transformation: epithelial-to-mesenchymal transition (EMT) or small cell lung cancer (SCLC) histological conversion [4,32,33,35,38,39]. See Table 1(B) for a summary of all major oncogenic drivers and key resistance mechanisms.

Bispecific antibodies (bsAbs) have recently emerged as a promising therapeutic approach to address these limitations. By simultaneously engaging two distinct antigens, bsAbs can overcome pathway redundancy, enhance receptor downregulation, and recruit immune effector cells [15,40,41]. Clinically, approved agents such as amivantamab (EGFR/MET) and zenocutuzumab (HER2/HER3) have demonstrated meaningful efficacy in specific molecular subsets of NSCLC, while a broad pipeline of investigational agents continues to expand [15,34,42].

This review examines the molecular rationale, clinical development, and therapeutic positioning of bsAbs in oncogene-driven NSCLC, with a focus on their comparative relationship to TKIs and antibody–drug conjugates, and their potential role within future treatment paradigms [34,40,41].

## 2. Molecular Basis of Bispecific Antibody Technology

### 2.1. Structural Designs of Bispecific Antibodies

Bispecific antibodies are engineered proteins capable of simultaneously binding two distinct antigens or epitopes. Over 100 structural formats have been described, broadly classified into IgG-like and non-IgG (fragment-based) formats based on the presence or absence of an Fc region [41,43,44]. See Table 2 for a comparative overview of relevant structural formats.

IgG-like asymmetric formats maintain the full structural scaffold of a natural immunoglobulin, with two distinct heavy-chain/light-chain pairs stabilized by various heterodimerization technologies such as the DuoBody platform (controlled Fab-arm exchange), knobs-into-holes CH3 engineering, and CrossMab domain swapping [43,44]. Amivantamab is produced via the DuoBody platform, enabling a fully human IgG1-like bispecific with an intact Fc region that supports antibody-dependent cellular cytotoxicity (ADCC) and antibody-dependent cellular phagocytosis (ADCP) [15]. Zenocutuzumab utilizes an IgG1-based CrossMab-related design that additionally incorporates Fc glycoengineering to enhance ADCC activity [15,45].

In contrast, non-IgG fragment-based formats such as bispecific T-cell engagers (BiTEs) and dual-affinity re-targeting (DART) proteins lack an Fc region, resulting in smaller molecular size and no Fc-mediated effector functions [43,44]. While their compact structure facilitates superior tissue penetration and potentially improved CNS access, they require more frequent dosing due to short serum half-lives unless modified with albumin-binding domains or Fc fusions [41,43]. While tarlatamab (DLL3/CD3 BiTE) represents the first FDA-approved BiTE-format bsAb in thoracic oncology, its indication is exclusively in SCLC; it is included as a structural exemplar. AMG 596 (EGFRvIII/CD3), an investigational BiTE in Phase I evaluation in EGFR-amplified solid tumors including NSCLC, represents an NSCLC-relevant example of this class [41,45].

### 2.2. Biological Rationale for Dual Receptor Targeting

The fundamental oncogenic rationale for dual-antigen targeting in NSCLC stems from the inherent redundancy and plasticity of receptor tyrosine kinase (RTK) signaling networks [4,41]. For example, EGFR and MET engage in ligand-independent transactivation, with MET amplification representing the most common measurable mechanism of acquired resistance to both first-generation and third-generation EGFR TKIs, accounting for 3–19% of osimertinib resistance cases [32,33]. Similarly, HER3 upregulation following EGFR inhibition maintains PI3K-AKT pathway activation through HER2-HER3 heterodimerization, creating a critical resistance bypass that single-agent EGFR TKIs cannot effectively block [33,41].

BsAbs address pathway redundancy in at least three mechanistic dimensions. First, by occupying both receptor extracellular domains simultaneously, they promote co-receptor crosslinking and receptor internalization/degradation, reducing total surface receptor density more effectively than single-target agents [15,45]. Second, bsAbs with an intact Fc region engage Fc-gamma receptors (FcγRs) on NK cells and macrophages, triggering ADCC and ADCP. Notably, amivantamab additionally mediates trogocytosis—the transfer of EGFR and MET fragments from tumor cells to monocytes—further depleting receptor levels on the tumor surface [14,34]. Third, the dual-targeting capability of bsAbs allows simultaneous recognition of heterogeneous tumor cell populations that co-express different receptor combinations, an important property in the context of clonal heterogeneity during acquired TKI resistance [34,41]. At the signaling level, EGFR/MET bispecifics converge on the RAS-MAPK and PI3K-AKT axes, producing more complete pathway suppression than single-receptor blockade [15].

### 2.3. Selectivity, Binding Affinity, and Tumor Microenvironment Considerations

BsAbs can be engineered with differential binding affinities for their two targets to optimize selective engagement of tumor cells over normal tissues [41,44]. Zenocutuzumab exploits higher HER2 surface density on tumor cells: its HER2-binding arm captures the antibody preferentially on HER2-overexpressing cancer cells, positioning the HER3-blocking arm to prevent NRG1-HER3 interaction, representing a mechanism termed ‘Dock & Block’ [15,45].

Key pharmacological limitations include molecular size (~150 kDa for IgG-like formats), which restricts CNS penetration relative to small-molecule TKIs (~400–700 Da) [15,44]. Tissue penetration into solid tumors may also be impaired by elevated interstitial pressure and a dense extracellular matrix. Furthermore, receptor expression heterogeneity across primary and metastatic sites may limit the predictive utility of single-site biopsies [15,41,44]. The tumor microenvironment can additionally modulate Fc receptor expression on effector cells, potentially attenuating ADCC activity in immunosuppressive contexts [41].

## 3. Clinical Development of Bispecific Antibodies in Oncogene-Driven NSCLC

### 3.1. EGFR/MET Bispecific Antibody: Amivantamab

Amivantamab is a fully human, IgG1-based EGFR/MET bispecific antibody produced via the DuoBody platform. It has received FDA approval for NSCLC harboring both classic EGFR mutations (exon 19 deletions and L858R) and non-classic mutations including exon 20 insertions. Its mechanisms of action include ligand binding prevention, receptor degradation through trogocytosis and internalization, and immune effector cell engagement via ADCC and ADCP, thereby addressing primary oncogenic signaling and resistance mechanisms such as MET amplification [15,43]. Phase I CHRYSALIS Trial: In patients with advanced NSCLC harboring EGFR exon 20 insertions who had progressed on platinum-based chemotherapy (*n* = 81), amivantamab monotherapy achieved an ORR of 40%, a mDOR of 11.1 months, and a mPFS of 8.3 months. The safety profile was characterized by rash (89%), IRRs (67%), and paronychia. These data supported FDA approval in the post-platinum setting in 2021 [15].

Phase III PAPILLON Trial: Among 308 treatment-naïvepatients with NSCLC harboring EGFR exon 20 insertions, amivantamab plus carboplatin-pemetrexed significantly improved mPFS (11.4 vs. 6.7 months; HR 0.40; 95% CI 0.30–0.53; *p* < 0.001) and ORR (73% vs. 47%) compared with chemotherapy alone. Grade ≥3 adverse events occurred in 75% of patients in the combination arm. These results established amivantamab–chemotherapy as the first-line standard of care for EGFR exon 20 insertion NSCLC, and supported FDA approval in 2024 [16].

Phase I/Ib CHRYSALIS-2 Trial: In 45 patients with EGFR-mutant NSCLC who had progressed on both osimertinib and platinum-based chemotherapy, amivantamab–lazertinib achieved an ORR of 28–35% and a mDOR of 8.3 months. Critically, responses were observed irrespective of the specific mechanism of osimertinib resistance, including in patients with MET amplification, secondary EGFR mutations, and EGFR/MET-independent resistance—demonstrating biomarker-agnostic activity [46].

Phase III MARIPOSA Trial: In 1074 patients with previously untreated EGFR-mutant (exon 19del/L858R) advanced NSCLC, amivantamab–lazertinib significantly prolonged mPFS compared with osimertinib (23.7 vs. 16.6 months; HR 0.70; 95% CI 0.58–0.85; *p* < 0.001). An updated overall survival (OS) analysis at a median follow-up of 37.8 months demonstrated a statistically significant OS benefit (HR 0.75; 95% CI 0.61–0.92; *p* = 0.005), establishing amivantamab–lazertinib as the first regimen to improve OS over osimertinib in first-line EGFR-mutant NSCLC [13,14]. Subgroup analyses confirmed benefit in high-risk populations including those with TP53 co-mutations, liver metastases, and detectable baseline ctDNA. Grade ≥3 adverse events occurred in 75% vs. 43% of patients; IRRs in 63% and VTEs in 37% were unique to the amivantamab-containing arm [13].

Phase III MARIPOSA-2 Trial: In 657 patients with EGFR-mutant NSCLC (exon 19del/L858R) who had progressed on osimertinib, amivantamab plus carboplatin-pemetrexed improved mPFS (6.3 vs. 4.2 months; HR 0.48; 95% CI 0.36–0.64; *p* < 0.001) and ORR (64% vs. 36%) compared with chemotherapy alone. Benefit was consistent across all resistance subgroups including MET amplification, secondary EGFR mutations (C797S), and EGFR/MET-independent resistance mechanisms, confirming the biomarker-agnostic utility of amivantamab in the post-osimertinib setting [47].

Pharmacological Comparison with TKIs: Administered intravenously with a half-life of ~14 days (q1–2 weeks dosing), amivantamab contrasts sharply with orally bioavailable daily TKIs in administration, onset of action, and toxicity profile. Amivantamab is associated with higher rates of IRRs, paronychia, dermatitis, and VTE compared to TKIs [13,15,47]. TKI resistance typically arises via secondary EGFR kinase mutations (C797S) and MET amplification, whereas amivantamab resistance arises via antigen escape (EGFR/MET downregulation), FcγR polymorphisms attenuating immune effector function, and compensatory RTK activation (HER3, AXL, IGF1R) [15,32,33].

### 3.2. HER2/HER3 Bispecific Antibody: Zenocutuzumab

Zenocutuzumab is a full-length, glycoengineered IgG1 bispecific antibody employing a ‘Dock & Block’ mechanism: the HER2-binding arm anchors the antibody to HER2-overexpressing tumor cells, while the HER3-blocking arm prevents neuregulin-1 (NRG1) binding, thereby blocking HER2-HER3 heterodimerization and downstream PI3K-AKT signaling [15,45]. This mechanism is specifically relevant in solid tumors harboring NRG1 gene fusions—potent oncogenic drivers present in ~1% of NSCLCs that activate HER3 signaling [42].

Phase I/II eNRGy Trial: In patients with NRG1 fusion-positive cancers (*n* = 111 NSCLC; *n* = 64 pancreatic adenocarcinoma), zenocutuzumab achieved an ORR of 34% by investigator assessment (29% by BICR) in NSCLC and 43% in pancreatic cancer, with a mDOR of 12.9 months and a mPFS of 11.0 months in NSCLC. NRG1 fusion status was determined by RNA-based next-generation sequencing, underscoring the requirement for comprehensive molecular profiling beyond standard DNA panels. The safety profile was favorable, with most adverse events being grade 1–2 and treatment discontinuation in <3% of patients. Based on these Phase II data, zenocutuzumab received FDA accelerated approval in December 2024 for advanced, unresectable, or metastatic NSCLC or pancreatic adenocarcinoma harboring NRG1 fusions after prior systemic therapy [42].

### 3.3. Other Bispecific Antibodies Under Development

Beyond EGFR/MET and HER2/HER3, a growing pipeline of investigational bsAbs has entered clinical evaluation. In a Phase I/IIa trial, AFM24—a bsAb targeting EGFR and CD16A on NK cells—demonstrated a disease control rate (DCR) of approximately 50% in heavily pretreated EGFR-mutant NSCLC [34]. SI-B001, an EGFR/HER3 bsAb, achieved an ORR of approximately 50% when combined with docetaxel in EGFR/ALK wild-type NSCLC patients who had failed prior anti-PD-1/PD-L1 therapy plus platinum chemotherapy [34].

BL-B01D1 is an innovative Phase I bispecific ADC combining an EGFR/HER3-targeting bsAb with a DXd cytotoxic payload. In a Phase I study, it achieved an ORR of 52.5% in EGFR-mutant NSCLC including patients resistant to third-generation EGFR TKIs, with a mPFS of 11.1 months in the TKI-resistant subgroup, representing a novel cross-class construct bridging bsAb and ADC modalities [34].

MCLA-129, a cMET/EGFR bispecific antibody, has demonstrated an ORR of 43.5% in Phase I/II studies in NSCLC harboring MET exon 14 skipping mutations, directly addressing the biological rationale for dual EGFR/MET co-targeting in this driver subset [34]. See Table 3 for a summary of early-phase and pivotal clinical trials of bsAb in NSCLC.

Expanding the bsAb pipeline beyond HER-family targets, investigational bsAbs are under development in non-EGFR driver subtypes: ALK/EGFR bispecifics targeting both the ALK fusion and EGFR bypass activation; RET/MET dual-targeting constructs addressing co-activation in RET-rearranged NSCLC; and bsAb combinations with KRAS G12C inhibitors to address bypass resistance. PD-L1/VEGF bispecifics (ivonescimab/HLX10 in non-driver NSCLC), DLL3/CD3 (tarlatamab, SCLC-approved), CLDN18.2/4-1BB (PM1032), and CLDN4/CD137 (ASP1002) further expand the conceptual scope of bsAb oncology [34,45].

### 3.4. Comparison with Antibody–Drug Conjugates (ADCs)

Antibody–drug conjugates (ADCs) have significantly broadened treatment options across multiple cancers and now represent an important therapeutic class in NSCLC [36]. Three ADCs have received FDA approval specifically for NSCLC: trastuzumab deruxtecan (T-DXd) for HER2-mutant NSCLC (August 2022), telisotuzumab vedotin for c-MET protein-overexpressing non-squamous NSCLC (May 2025), and datopotamab deruxtecan (Dato-DXd) for EGFR-mutant pretreated NSCLC (June 2025) [28,48,49,50].

In the Phase II DESTINY-Lung01 trial, T-DXd achieved an ORR of 55% in HER2-mutant NSCLC, with a mOS of 17.8 months [48]. The confirmatory Phase II DESTINY-Lung02 trial demonstrated an ORR of 49.0%, a mPFS of 9.9 months, and a mOS of 19.5 months, with a reduced rate of interstitial lung disease (ILD) of 12.5% (grade ≥3: 1.6%) compared with 26% observed in DESTINY-Lung01 [28]. Collectively, these Phase II trials established T-DXd as the preferred therapy for HER2-mutant NSCLC and prompted accelerated FDA approval [28,48].

In the Phase III TROPION-Lung01 trial, Dato-DXd was compared with docetaxel in previously treated patients with advanced NSCLC. Dato-DXd improved mPFS (4.4 vs. 3.7 months; HR 0.75) and demonstrated a more favorable toxicity profile than docetaxel, with a lower rate of grade ≥3 treatment-related adverse events [49]. On the basis of subset analyses demonstrating superior benefit in EGFR-mutant NSCLC, Dato-DXd received FDA approval specifically for this molecular subset in June 2025 [49].

In the Phase II LUMINOSITY trial, telisotuzumab vedotin, an anti-c-MET ADC conjugated to monomethyl auristatin E (MMAE), achieved an ORR of 35% in c-MET-overexpressing non-squamous NSCLC, with a mPFS of 5.4 months and a mOS of 14.5 months [50]. Grade ≥3 peripheral neuropathy was observed in 7% of patients, and a manageable rate of cytopenias was reported. These Phase II data supported FDA approval in May 2025 [50].

Patritumab deruxtecan (HER3-DXd), a HER3-targeting ADC with a DXd payload, has also demonstrated encouraging Phase I/II activity in EGFR-mutant NSCLC after osimertinib failure, with an ORR of approximately 39% and a mPFS of 8.2 months, establishing HER3 as a tractable target in the post-osimertinib resistance setting [36]. See Table 4(A,B) for a summary of key ADC clinical trial data.

Mechanistically, ADCs differ fundamentally from bsAbs: they consist of a monoclonal antibody conjugated to a cytotoxic payload via a chemical linker, enabling targeted intracellular drug delivery without the requirement for Fc-mediated effector recruitment [36]. ADCs therefore do not produce the ADCC/ADCP-mediated immune effects seen with Fc-containing bsAbs. From a safety standpoint, bsAbs are associated with higher rates of immune-mediated, hematologic, and dermatologic toxicities, while ADCs carry risks of ILD, cytopenias, and peripheral neuropathy [28,36,48,49,50]. Resistance to bsAbs arises via antigen escape, Fc receptor polymorphisms, and compensatory RTK activation, whereas ADC resistance involves antigen loss, impaired internalization, lysosomal dysfunction, and drug efflux [15,36,41]. With their distinct mechanisms and toxicity profiles, bsAbs and ADCs offer complementary therapeutic opportunities that may be leveraged in sequential or combined strategies. See Table 5 for a comparative analysis of bsAbs and ADCs.

## 4. Positioning Bispecific Antibodies in the Treatment Landscape

### 4.1. Advantages of Bispecific Antibodies over TKIs

BsAbs offer distinct advantages compared to TKIs in the treatment landscape of oncogene-driven NSCLC (see Table 6) [15,41]. Most critically, their dual-targeting capability enables simultaneous blockade of multiple receptor pathways, directly addressing the resistance mechanisms that undermine TKI monotherapy. Third-generation EGFR TKIs such as osimertinib and lazertinib are small-molecule inhibitors that competitively bind the ATP-binding pocket of mutant EGFR, blocking downstream signaling [3,32]. While osimertinib achieved a landmark mPFS of 18.9 months in the Phase III FLAURA trial as first-line monotherapy, resistance emerges through diverse mechanisms, most commonly MET amplification and secondary EGFR mutations, within 18–24 months [7]. The dual EGFR/MET targeting of amivantamab directly addresses MET-driven TKI resistance, and since bsAb activity does not depend on the integrity of the kinase active site, bsAbs remain active across diverse resistance genotypes [15,32,33].

BsAbs can simultaneously engage heterogeneous tumor cell populations expressing different receptor combinations, a biologically critical property in the context of clonal evolution during TKI pressure [34,41]. In the Phase III MARIPOSA trial, amivantamab–lazertinib maintained efficacy across diverse molecular subgroups including those with TP53 co-mutations and detectable ctDNA, adverse prognostic features associated with inferior outcomes on osimertinib monotherapy [13]. Similarly, in the Phase III MARIPOSA-2, amivantamab–chemotherapy improved mPFS over chemotherapy across all pre-specified resistance subgroups including MET amplification, secondary EGFR mutations, and EGFR/MET-independent resistance mechanisms [47].

BsAbs with intact Fc regions additionally recruit immune effector cells via FcγR interactions or direct CD3 engagement, facilitating immune-mediated tumor cell death—a biological dimension entirely absent from small-molecule TKIs [15,41]. This immune-engaging mechanism may contribute to deeper and more durable responses, particularly in tumors with residual immune infiltration [34,41].

### 4.2. Limitations Compared with TKIs

BsAbs are associated with a distinct and clinically significant toxicity profile that markedly differs from that of TKIs. In the Phase III MARIPOSA trial, grade ≥3 adverse events occurred in 75% of patients receiving amivantamab–lazertinib versus 43% with osimertinib, with IRRs (63% vs. 0%) and VTEs (37% vs. 9%) notably more frequent with amivantamab [13]. Dose reductions and treatment discontinuations due to adverse events were also more frequent with amivantamab-based regimens, raising important quality-of-life considerations [13,47].

Amivantamab requires intravenous administration, often on consecutive infusion days during cycle 1, representing a significant logistical and patient burden compared to daily oral TKI therapy [15]. Additionally, pharmacodynamic onset is inherently slower for immune-engaging bsAbs compared to the rapid biochemical responses achieved by small-molecule TKIs [15].

The large molecular size of IgG-like bsAbs (~150 kDa for amivantamab) relative to small-molecule TKIs (~400–700 Da) has been proposed as a theoretical pharmacological barrier to blood–brain barrier penetration [15,44]. However, it is important to distinguish this theoretical, size-based concern from the actual clinical CNS outcomes observed with amivantamab-based regimens, which do not support a meaningful intracranial efficacy deficit.

In the Phase III MARIPOSA trial, all patients underwent protocol-mandated serial brain MRI every 8 weeks for the first 3 years (then every 12 weeks), providing one of the most rigorous prospective intracranial datasets available in EGFR-mutant NSCLC [13]. Intracranial response rates were comparable between arms (intracranial ORR approximately 77% with both amivantamab–lazertinib and osimertinib), and intracranial PFS numerically favored the amivantamab–lazertinib combination, with a hazard ratio of 0.69–0.79 across reported analyses. At the 36-month landmark, 36–38% of patients receiving amivantamab–lazertinib remained free of intracranial progression compared with 18% receiving osimertinib monotherapy. Intracranial duration of response was also longer with the combination (median not reached vs. 24.4 months with osimertinib), and 51% of intracranial responders to amivantamab–lazertinib maintained that response at 3 years, compared with 0% of osimertinib responders. Similar intracranial PFS benefit was independently observed with amivantamab–chemotherapy (without lazertinib) in the post-osimertinib MARIPOSA-2 trial, further supporting that amivantamab itself contributes meaningfully to CNS disease control rather than CNS benefit being attributable to a TKI partner alone [13,47].

The mechanism underlying this unexpectedly robust intracranial activity is not fully established. Rather than a simple ‘division of labor’ in which lazertinib alone accounts for CNS control, the published MARIPOSA-2 analysis explicitly notes that the mechanism by which amivantamab improves intracranial PFS may be direct or immune-mediated, and it remains an open question warranting further investigation.

Clinically, these data have practical implications: amivantamab–lazertinib is recognized as an NCCN-preferred combination option for EGFR-mutant NSCLC with brain metastases, on par with brain-penetrant TKI monotherapy. Treatment selection in patients with CNS disease should therefore be guided primarily by patient-specific factors such as VTE/bleeding risk and tolerance for IV administration rather than by an assumption of inferior CNS efficacy with bsAb-based regimens.

### 4.3. Bispecific Antibodies in the Post-TKI Resistance Setting

The most established clinical role for bsAbs in the current landscape is in the post-TKI resistance setting, where the molecular complexity of acquired resistance limits the efficacy of single-target strategies [15,51]. Amivantamab–chemotherapy demonstrated meaningful clinical benefit in patients with classic EGFR-mutant NSCLC after osimertinib failure in the Phase III MARIPOSA-2 trial, providing a viable option for a population with limited alternatives and demonstrating biomarker-agnostic activity across diverse resistance mechanisms [47].

Emerging data suggests a potential role for bsAbs in KRAS G12C and MET-amplified acquired resistance settings, where single-agent TKI approaches are constrained by the complexity of resistance networks [34,45]. Sequential strategies (TKI → bsAb → TKI or TKI → bsAb-chemotherapy) represent an active area of clinical investigation. The PALOMA-3 trial is evaluating a subcutaneous formulation of amivantamab to reduce the administration burden and improve the tolerability profile [34,45].

### 4.4. Pivotal Trials: FLAURA vs. FLAURA 2 vs. MARIPOSA

Three pivotal Phase III trials have defined first-line treatment intensification strategies in EGFR-mutant NSCLC: FLAURA (osimertinib monotherapy vs. first-generation TKI), FLAURA2 (osimertinib plus chemotherapy vs. osimertinib), and MARIPOSA (amivantamab–lazertinib vs. osimertinib). While FLAURA2 and MARIPOSA share a first-line intensification philosophy, they differ fundamentally in mechanism: FLAURA2 intensifies cytotoxic killing through chemotherapy, while MARIPOSA introduces biological dual-targeting with immune engagement. Table 7 provides a structured analysis of the trials [7,8,13,14].

## 5. Mechanisms of Response and Resistance to Bispecific Antibodies

### 5.1. Predictors of Response

Predictive biomarkers for bsAb activity in NSCLC remain an active area of clinical and translational investigation [15,34]. For amivantamab, receptor expression levels of EGFR and MET protein by immunohistochemistry (IHC) have been evaluated as potential correlates of response, though validated quantitative thresholds for clinical patient selection have not yet been established [15].

Circulating tumor DNA (ctDNA) allele fraction and co-mutation profiles have emerged as increasingly informative biomarkers. In the Phase III MARIPOSA trial, high ctDNA burden at baseline was associated with inferior outcomes on osimertinib monotherapy, whereas amivantamab–lazertinib maintained efficacy in this adverse molecular context [13]. These findings suggest that bsAbs may provide particular benefit in patients with high-risk molecular features including TP53, STK11, and KEAP1 co-mutations, which are associated with reduced immunogenicity and accelerated clonal evolution [13,34].

For zenocutuzumab, NRG1 fusion status, detectable by RNA-based next-generation sequencing, is the definitive biomarker for patient selection, underscoring the importance of comprehensive molecular profiling over sole reliance on histological classification [34,42].

### 5.2. Resistance Mechanisms Unique to Bispecific Antibodies

Resistance mechanisms to bsAbs are mechanistically distinct from those governing TKI failure and represent an evolving area of investigation [15,41]. The primary class of bsAb resistance involves reduction or loss of target receptor expression on tumor cells (antigen escape), which eliminates the binding substrate for both the therapeutic and immune effector arms of the bispecific [15,34]. Fc receptor polymorphisms, particularly in FcγRIIIA (CD16A), can attenuate ADCC efficacy by impairing NK cell engagement. Compensatory upregulation of alternative RTKs—including HER3, IGF1R, and AXL—can re-activate downstream MAPK and PI3K-AKT pathways, bypassing dual EGFR/MET inhibition [15,41]. Finally, downstream MAPK pathway reactivation via acquired KRAS, BRAF, or NRAS mutations can render tumor cells independent of upstream receptor-level blockade [15,33].

### 5.3. Overcoming Resistance to Bispecific Antibodies

The most rational strategy for overcoming bsAb resistance is combination with TKIs to simultaneously address kinase-dependent escape pathways. Amivantamab–lazertinib is the prototypical example, where the TKI component suppresses EGFR kinase activity while amivantamab blocks EGFR/MET receptor function and recruits immune effectors [13,34]. This mechanistic synergy provides a rationale for bsAb-TKI combinations in both frontline and post-progression settings [13,51].

Bispecific + ADC combinations represent a complementary resistance-overcoming strategy, leveraging the cytotoxic payload delivery of ADCs alongside the immune engagement of bsAbs. The emerging class of bispecific ADCs, exemplified by BL-B01D1 (EGFR/HER3 + DXd), represents a novel construct designed to simultaneously address multiple resistance mechanisms within a single molecule [34,36].

Immunotherapy partnerships, pairing immune-engaging bsAbs with PD-1/PD-L1 checkpoint inhibitors, may potentiate durable antitumor immunity in tumors with residual immunogenicity, particularly in the post-TKI setting where immune exhaustion may be partially reversible. The rationale for triplet regimens (TKI + bsAb + IO) is being explored in early-phase studies, with toxicity management remaining a critical clinical challenge [34,41,45].

## 6. Will Bispecific Antibodies Displace TKIs or Complement Them?

The central question of whether bsAbs will replace or complement TKIs in oncogene-driven NSCLC can be addressed through the current weight of clinical evidence. Multiple lines of data converge to suggest that outright displacement is unlikely in the near to medium term [15,34,41].

TKIs retain important advantages for rapid cytoreduction, particularly in patients with high disease burden, symptomatic presentation, or disease requiring urgent tumor control [3]. Third-generation TKIs achieve rapid, profound, and biochemically measurable responses within days of initiation [3,6,7].

Conversely, bsAbs excel in settings where dual receptor pathway blockade and immune activation are therapeutically decisive. The Phase III MARIPOSA trial established that amivantamab–lazertinib outperforms osimertinib monotherapy in patients with high-risk molecular features, defining a clinically actionable complementary paradigm where bsAb addition to a TKI provides incremental benefit over TKI alone [13].

At present, the evidence strongly supports a complementary model. Scenarios where bsAbs are preferred and TKIs have limited activity: (1) EGFR exon 20 insertion mutations, where TKIs are insufficiently active and amivantamab (with or without chemotherapy) is now standard of care; (2) NRG1 fusion-positive NSCLC, where no TKI has activity and zenocutuzumab is the only FDA-approved targeted option; and (3) post-osimertinib acquired resistance, where amivantamab–chemotherapy provides biomarker-agnostic benefit over chemotherapy alone [15,16,42,47].

Scenarios where bsAbs complement rather than replace TKIs: (1) first-line EGFR classical mutations in high-risk subgroups, where amivantamab–lazertinib improves on osimertinib monotherapy but does not eliminate the need for TKI activity; and (2) METex14 skipping mutations, where investigational bsAbs target EGFR/MET bypass but no approved bsAb yet exists for this indication [13,34].

Scenarios where TKIs remain clearly preferred: (1) non-EGFR driver subtypes (ALK, ROS1, RET, KRAS G12C) where no approved bsAb exists; and (2) patients unable to tolerate IV infusion or with high VTE risk. Notably, CNS-predominant EGFR-mutant disease is not among these scenarios: MARIPOSA intracranial data support amivantamab–lazertinib as an evidence-based option alongside brain-penetrant TKI monotherapy rather than a setting where bsAbs are disadvantaged [3,13].

**Figure 1:**
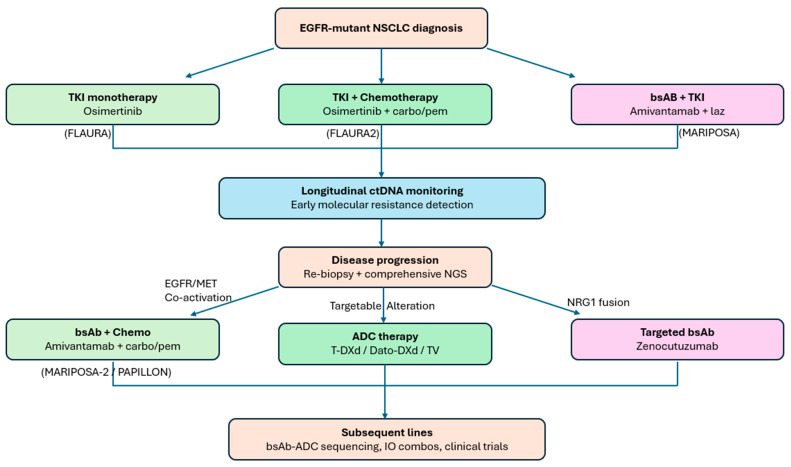
Proposed therapeutic sequencing algorithm for EGFR-mutant advanced NSCLC integrating bispecific antibodies. Three first-line options are presented without implied hierarchy: TKI monotherapy (osimertinib; FLAURA), TKI plus chemotherapy (osimertinib + carboplatin/pemetrexed; FLAURA2), and bispecific antibody plus TKI (amivantamab + lazertinib; MARIPOSA). Longitudinal ctDNA monitoring guides early resistance detection. Upon progression, rebiopsy with comprehensive next-generation sequencing informs second-line selection: bsAb-based chemotherapy combinations (MARIPOSA-2/PAPILLON), ADC therapy (T-DXd, Dato-DXd, or telisotuzumab vedotin), or targeted bsAbs (zenocutuzumab for NRG1 fusions). ADC, antibody–drug conjugate; bsAb, bispecific antibody; carbo/pem, carboplatin/pemetrexed; ctDNA, circulating tumor DNA; laz, lazertinib; NGS, next-generation sequencing; TKI, tyrosine kinase inhibitor; TV, telisotuzumab vedotin.

For future therapeutic sequencing, an emerging paradigm may involve: (1) TKI monotherapy for initial rapid disease control; (2) bsAb-TKI combination upon ctDNA detection of early resistance clones; (3) bsAb-chemotherapy for overt acquired resistance; and (4) ADC-based therapy for subsequent progression [13,34,41,47]. See Figure 1 for a proposed therapeutic sequencing algorithm integrating bsAbs in EGFR-mutant advanced NSCLC. As longer-term OS data from MARIPOSA and related trials mature, and as subcutaneous bsAb formulations reduce administration burden, combination-based front-line strategies incorporating bsAbs are likely to achieve broader regulatory and clinical acceptance [13,51].

## 7. Future Directions

The field of bsAbs in NSCLC is poised for rapid evolution across engineering, clinical, and translational dimensions. A priority engineering challenge is improving CNS penetration, which may be addressed through Fc-fused albumin binding of fragment-based formats, development of blood–brain barrier-crossing bsAb platforms, or rational combination with established CNS-active TKIs [34,41,44].

Strategies to mitigate infusion-related toxicity and improve tolerability are urgently needed to facilitate broader clinical adoption. The PALOMA-3 trial demonstrated that subcutaneous amivantamab administration significantly reduced the rate of IRRs compared with intravenous delivery, while the COCOON trial is evaluating prophylactic dermatologic management protocols in the context of amivantamab–lazertinib, suggesting that proactive supportive care strategies can meaningfully mitigate bsAb-specific toxicities [34,45].

Molecular biomarker development represents perhaps the highest-priority translational objective. Prospective studies defining quantitative EGFR and MET IHC expression thresholds, ctDNA allele fraction cutoffs, and co-mutation profiles (TP53, STK11, KEAP1) that predict differential benefit from bsAb versus TKI strategies will be essential for rational, personalized patient selection [13,34].

On the engineering frontier, trispecific antibodies targeting three distinct antigens simultaneously represent the next conceptual evolution of multifunctional oncology biologics. By simultaneously engaging a tumor antigen, an immune effector receptor (e.g., CD3 or CD16A), and a co-stimulatory molecule, trispecifics aim to maximize immune synapse formation while minimizing systemic cytokine release [41].

Integration with ctDNA-guided adaptive therapy frameworks, wherein treatment modifications are triggered by molecular evidence of emerging resistance rather than radiographic progression, could enable earlier and more targeted deployment of bsAbs or bsAb-TKI combinations at a stage where disease burden remains manageable and immune function is preserved [34,41].

Randomized trials directly comparing TKI monotherapy versus bsAb monotherapy in first-line settings for specific molecular subsets are needed to define the intrinsic standalone clinical value of bispecific antibodies independent of combination strategies. The potential for bsAb-TKI doublets to become standard first-line therapy in high-risk EGFR-mutant NSCLC is likely to gain further traction as long-term OS data from the MARIPOSA study mature [13,34,51].

## 8. Conclusions

Bispecific antibodies represent a meaningful therapeutic evolution in oncogene-driven NSCLC, offering a mechanistically distinct approach that integrates dual receptor blockade with immune effector engagement to overcome the inherent limitations of TKI monotherapy [15,41]. Clinical data for amivantamab and zenocutuzumab demonstrate meaningful activity across both treatment-naïve and TKI-resistant settings, particularly in molecularly defined subgroups including EGFR exon 20 insertions and NRG1 fusions [13,16,42,47].

However, current limitations, including increased immune-mediated and thromboembolic toxicity, the requirement for intravenous administration, restrict their ability to replace TKIs as standard first-line monotherapy [13,15]. The available evidence instead strongly supports a complementary role, particularly in combination with TKIs or chemotherapy and in the management of acquired resistance [13,47,51].

Continued translational research is essential to define optimal treatment sequencing strategies, validate predictive biomarkers, and develop next-generation bsAb constructs with improved tolerability [34,41]. As these advances converge, bispecific antibodies are poised to assume an increasingly central and potentially practice-defining role within combination-based therapeutic paradigms for oncogene-driven NSCLC [15,34,41].

## Figures and Tables

**Table 1:** (A) Oncogenic Drivers in NSCLC: Prevalence, Typical Population, and Key Clinical Trial Data. (B) Drug-Specific and Emerging Resistance Mechanisms in Oncogene-Driven NSCLC.

**(A)**						
**Driver**	**Prevalence**	**Typical Population**	**Agent**	**Clinical Trial (Phase)**	**Median PFS**	**Ref.**
EGFR Exon 19 deletion/L858R	~18–51% (global)	Female, never-smoker, Asian	Osimertinib	FLAURA (III)	18.9 mo	[7]
			Osimertinib + carbo/pem	FLAURA2 (III)	25.5 mo	[8]
			Gefitinib	IPASS (III)	5.7 mo	[9]
			Erlotinib	EURTAC (III)	9.7 mo	[10]
			Dacomitinib	ARCHER 1050 (III)	14.7 mo	[11]
			Afatinib	LUX-Lung 3 (III)	11.1 mo	[12]
			Amivantamab + Lazertinib	MARIPOSA (III)	23.7 mo	[13,14]
EGFR Ex20 insertion	2–3% of EGFR mutations	Female, never-smoker	Amivantamab (bsAb)	CHRYSALIS (I)	8.3 mo	[15]
			Amivantamab + chemo	PAPILLON (III)	11.4 mo	[16]
ALK rearrangement	3–7%	Young, never-smoker	Alectinib	ALEX (III)	34.8 mo	[17]
			Ceritinib	ASCEND-4 (III)	16.6 mo	[18]
			Ensartinib	eXALT3 (III)	25.8 mo	[19]
			Brigatinib	ALTA-1L (III)	24.0 mo	[20]
			Lorlatinib	CROWN (III)	33.2+ mo (NR)	[21]
			Crizotinib	PROFILE 1014 (III)	10.9 mo	[22]
ROS1 rearrangement	1–2%	Young, female, never-smoker	Crizotinib	PROFILE 1001 ROS1 cohort (I)	19.2 mo	[23]
			Repotrectinib	TRIDENT-1 (I/II)	35.7 mo (treatment-naïve)	[24]
			Taletrectinib	TRUST-I (II)	49.6 mo (treatment-naïve)	[25]
			Entrectinib	STARTRK-2 (II)	15.7 mo	[6]
RET rearrangement	1–2%	Young, never-smoker	Selpercatinib	LIBRETTO-001 (I/II)	22.0 mo	[3]
			Pralsetinib	ARROW (I/II)	13.0 mo	[3]
METex14 skipping	3–4%	Older, mixed smoking	Tepotinib	VISION (II)	12.0 mo	[3]
			Capmatinib	GEOMETRY-mono-1 (II)	12.4 mo	[3]
KRAS G12C	~13% Western; lower Asian	Smokers, Caucasian	Sotorasib	CodeBreak 200 (III)	6.0 mo	[3]
			Adagrasib	KRYSTAL-12 (III)	5.4 mo	[3]
HER2 mutation/amplification	1.5–3%	Female, never-smoker	Zongertinib	Beamion LUNG-1 (Ia/Ib)	14.4 mo (treatment-naïve)	[26]
			Sevabertinib	SOHO-1 (I/II)	13.5 mo (treatment-naïve)	[27]
			T-DXd	DESTINY-Lung02 (II)	9.9 mo	[28]
BRAF V600E	~1–3%	Female, never/light smoker	Dabrafenib + Trametinib	BRF113928 (II)	10.2 mo	[29]
			Encorafenib + Binimetinib	PHAROS (II)	30.2 mo (treatment-naïve)	[30]
NTRK1/2/3 fusion	<1%	Young; No sex/smoking predominance	Larotrectinib	Pooled Phase I/II	28.3 mo	[31]
			Entrectinib	STARTRK pooled (I/II)	15.7 mo	[6]
**(B)**						
**Driver**	**On-Target (Secondary Kinase Mutations)**	**Off-Target Bypass Activation**		**Downstream Signaling Alterations**	**Phenotypic/Epigenetic**	**Ref.**
EGFR Exon 19 deletion/L858R	C797S, L718Q/V, G796R/S; compound mutations C797S + T790M	MET amplification (3–19%), HER2 amplification, AXL/GAS6 overexpression, FGFR amplification, PIK3CA mut, RET fusions, YAP1/TEAD activation		KRAS G12C/V, NRAS mut, BRAF class II fusions, MEK1/2 mutations, NF1 loss, CDKN2A loss	EMT, SCLC transformation (~5%), cancer stem cell phenotype, SWI/SNF mutations	[4,32,33]
EGFR Ex20 insertion	Secondary EGFR mutations (rare); amivantamab: antigen escape (EGFR/MET downregulation)	Bypass via HER3, FGFR, AXL; MET-independent bypass activation		KRAS/BRAF/PI3K downstream reactivation	Partial EMT	[15,34]
ALK rearrangement	ALK G1202R (lorlatinib), L1196M, G1269A, I1171N/T, compound mutations	MET amplification, EGFR activation, KRAS mutation, SRC upregulation		KRAS/NRAS, NF1 loss, MAPK reactivation	EMT, tumor heterogeneity	[4,35]
ROS1 rearrangement	G2032R (solvent-front), L2026M, D2033N, S1986Y/F	MET amplification, KRAS activation, BRAF co-mutation		KRAS/MAPK downstream reactivation	EMT	[4,35]
RET rearrangement	G810R/S/C (solvent-front), V738A, L730V	KRAS mut, MET amplification, EMT-driven bypass		MAPK/PI3K reactivation	EMT	[35]
METex14 skipping	Secondary MET kinase mutations (D1228N/H/V, Y1230H/S/C)	EGFR activation, HER2/HER3 bypass, KRAS mut		PI3K/AKT/mTOR, MAPK reactivation	EMT	[4,33]
KRAS G12C	KRAS Y96D, H95R/Q (covalent binding site)	MET amplification, HER2/EGFR bypass, FGFR3 activation, SHP2 reactivation		MAPK reactivation, PI3K activation, CDK4/6-RB bypass	STK11/KEAP1 co-mutations, SCLC transformation	[4,35]
HER2 mutation/amplification	No classic secondary resistance kinase mutations described	HER3 upregulation, PI3K/AKT activation, EGFR bypass		PIK3CA mutations, PTEN loss, downstream PI3K/AKT	EMT, clonal selection under payload pressure	[33,36]
BRAF V600E	BRAF class switch (to class I homodimer)	EGFR reactivation, RAS mutations (NRAS, KRAS)		MEK/ERK reactivation, PI3K/AKT bypass	EMT, histological transformation	[4,35]
NTRK fusion	G595R, G667C, F589L (solvent-front)	RAS/MAPK bypass, MET amplification		MAPK reactivation, PI3K/AKT activation	EMT	[4,35]

ALK: anaplastic lymphoma kinase; bsAb: bispecific antibody; carbo/pem: carboplatin/pemetrexed; EGFR: epidermal growth factor receptor; Ex19del: exon 19 deletion; Ex20ins: exon 20 insertion; HER2: human epidermal growth factor receptor 2; KRAS: Kirsten rat sarcoma viral oncogene; L858R: leucine-858-arginine substitution; MET/METex14: mesenchymal–epithelial transition proto-oncogene/MET exon 14 skipping; mo: months; NR: not reached; NTRK: neurotrophic tyrosine receptor kinase; PFS: progression-free survival; RET: rearranged during transfection; ROS1: c-ros oncogene 1; T-DXd: trastuzumab deruxtecan; V600E: valine-600-glutamic acid substitution. AKT: protein kinase B; AXL: AXL receptor tyrosine kinase; BRAF: v-Raf murine sarcoma viral oncogene homolog B; CDK4/6-RB: cyclin-dependent kinase 4/6-retinoblastoma protein pathway; CDKN2A: cyclin-dependent kinase inhibitor 2A; EMT: epithelial-to-mesenchymal transition; ERK: extracellular signal-regulated kinase; FGFR(3): fibroblast growth factor receptor (3); GAS6: growth arrest-specific 6; HER2/HER3: human epidermal growth factor receptor 2/3; KEAP1: Kelch-like ECH-associated protein 1; MAPK: mitogen-activated protein kinase; MEK1/2: mitogen-activated protein kinase kinase 1/2; mTOR: mechanistic target of rapamycin; NF1: neurofibromin 1; NRAS: neuroblastoma RAS viral oncogene homolog; PI3K: phosphoinositide 3-kinase; PIK3CA: phosphatidylinositol-4,5-bisphosphate 3-kinase catalytic subunit alpha; PTEN: phosphatase and tensin homolog; RAS: rat sarcoma viral oncogene homolog; SCLC: small cell lung cancer transformation; SHP2: Src homology region 2 domain-containing phosphatase-2; SRC: proto-oncogene tyrosine-protein kinase Src; STK11: serine/threonine kinase 11; SWI/SNF: SWItch/Sucrose Non-Fermentable chromatin remodeling complex; TEAD: TEA domain transcription factor; YAP1: Yes-associated protein 1.

**Table 2:** Structural Formats of Bispecific Antibodies Relevant to Thoracic Oncology.

Format Class	Representative Example(s)	Structural Features	Fc-Mediated Effector Function
IgG-like Asymmetric (DuoBody)	Amivantamab	Dual Fab arms, intact IgG1 Fc; heterodimeric HC via controlled Fab-arm exchange	Full ADCC, ADCP, FcRn recycling
IgG-like Asymmetric (CrossMab)	Zenocutuzumab	Fab domain exchange to prevent LC mispairing; intact IgG1 Fc; glycoengineered	ADCC enhanced by glycoengineering
Fc-silent IgG-like	Various investigational agents	Engineered Fc with FcγR -silencing mutations	Minimal ADCC/CDC; reduced cytokine release
Fragment-based Non-IgG (BiTE)	AMG 596 (EGFRvIII/CD3, NSCLC Phase I); Tarlatamab (DLL3/CD3) *	Two scFv linked via flexible glycine-serine peptide; no Fc region	No Fc effectors; direct T-cell engagement via CD3; CRS risk
Non-IgG (DART)	Various preclinical NSCLC constructs	Disulfide-stabilized diabody; dual-affinity re-targeting	No Fc; direct immune cell recruitment

* Tarlatamab is included as a structural representative of the non-IgG BiTE format; while it is FDA approved for SCLC, no clinical NSCLC-specific efficacy data are currently available. ADCC: antibody-dependent cell cytotoxicity; ADCP: antibody-dependent cellular phagocytosis; CRS: cytokine release syndrome; scFv: single-chain variable fragment.

**Table 3:** (A) Early-Phase (Phase I/Ib) Clinical Trials of Bispecific Antibodies in NSCLC. (B) Pivotal (Phase II/III) Clinical Trials of Bispecific Antibodies in NSCLC.

**(A)**						
**Trial [Ref.]**	**Agent(s)**	**Phase**	**Population**	**ORR**	**Median PFS/DOR**	**Key Safety**
CHRYSALIS [15]	Amivantamab mono	I	EGFR Ex20ins, post-platinum (*n* = 81)	40%	PFS 8.3 mo; DOR 11.1 mo	Rash 89%, IRR 67%, paronychia
CHRYSALIS-2 Cohort A [46]	Amivantamab + lazertinib	I/Ib	EGFR classic mut, post-osimertinib + chemo (*n* = 45)	28–35%	DOR 8.3 mo	Rash, paronychia; manageable
AFM24 Phase I/IIa [34]	AFM24 (EGFR/CD16A)	I/IIa	EGFR-mutant NSCLC, heavily pretreated	DCR 50%	NR	IRR, rash; manageable
BL-B01D1 Phase I [34]	BL-B01D1 (EGFR/HER3 bsAb-ADC)	I	EGFR-mutant NSCLC, TKI-resistant (3rd-gen)	52.5%	mPFS 11.1 mo	Grade ≥3 TRAEs 67%; leukopenia
MCLA-129 Phase I/II [34]	MCLA-129 (cMET/EGFR)	I/II	METex14 skipping NSCLC	43.5%	Ongoing	IRR, peripheral edema
**(B)**						
**Trial [Ref.]**	**Agent(s)**	**Phase**	**Population**	**ORR**	**mPFS (HR; *p*-Value)**	**Key Safety**
PAPILLON [16]	Amivantamab + carbo/pem vs. chemo	III	EGFR Ex20ins, 1st-line (*n* = 308)	73% vs. 47%	11.4 vs. 6.7 mo (HR 0.40; *p* < 0.001)	Grade ≥3 AEs 75%; IRR 46%
MARIPOSA [13]	Amivantamab + lazertinib vs. osimertinib	III	EGFR Exon 19 deletion/L858R, 1st-line (*n* = 1074)	86% vs. 85%	23.7 vs. 16.6 mo (HR 0.70; *p* < 0.001); OS HR 0.75 [14]	Grade ≥3 AEs 75% vs. 43%; IRR 63%; VTE 37%
MARIPOSA-2 [47]	Amivantamab + carbo/pem ( + -laz) vs. chemo	III	EGFR Exon 19 deletion/L858R, post-osimertinib (*n* = 657)	64% vs. 36%	6.3 vs. 4.2 mo (HR 0.48; *p* < 0.001)	VTE 36%; Grade ≥3 AEs 72%
eNRGy [42]	Zenocutuzumab	I/II	NRG1 fusion+ NSCLC & pancreatic (*n* = 111 NSCLC)	34% (investigator); 29% (BICR) NSCLC	DOR 12.9 mo; mPFS 11.0 mo	Grade 1–2 predominant; Grade ≥3: 3%

DCR: disease control rate; DOR: duration of response; NR: not reported; TRAEs: treatment-related adverse events; VTE: venous thromboembolism; IRR: infusion-related reaction; BICR: blinded independent central review;; carbo/pem: carboplatin/pemetrexed.

**Table 4:** (A) Approved ADC Clinical Trials in NSCLC (Phase II/III). (B) Investigational ADC Clinical Trials in NSCLC (Phase I/II).

**(A)**						
**Trial [Ref.]**	**Agent**	**Phase**	**Population**	**ORR**	**Median PFS/OS**	**Key Safety**
DESTINY-Lung01 [48]	T-DXd (HER2)	II	HER2-mutant NSCLC, >=1 prior Tx (*n* = 91)	55%	mPFS 8.2 mo; mOS 17.8 mo	ILD 26% (Grade ≥3: 5%); nausea 74%
DESTINY-Lung02 [28]	T-DXd (HER2)	II	HER2-mutant NSCLC, >=1 prior Tx (*n* = 102)	49%	mPFS 9.9 mo; mOS 19.5 mo	ILD 12.5% (Grade ≥3: 1.6%); nausea 66%
TROPION-Lung01 [49]	Dato-DXd (TROP2) vs. docetaxel	III	Pretreated advanced NSCLC (*n* = 603)	26.4% vs. 12.8%	PFS 4.4 vs. 3.7 mo (HR 0.75; *p* = 0.004)	ILD 8.8% (Grade ≥3: 1.3%); stomatitis 42%
LUMINOSITY [50]	Telisotuzumab vedotin (c-MET)	II	c-MET overexpressing non-sq NSCLC (*n* = 148)	35%	mPFS 5.4 mo; mOS 14.5 mo	Periph neuropathy Grade ≥3: 7%; fatigue
**(B)**						
**Trial [Ref.]**	**Agent**	**Phase**	**Population**	**ORR**	**Median PFS/OS**	**Key Safety**
HERTHENA-Lung01 [36]	Patritumab deruxtecan (HER3-DXd)	I/II	EGFR-mutant NSCLC, post-osimertinib (*n* = 225)	39%	mPFS 8.2 mo; mOS 15.1 mo	ILD 13% (Grade ≥3: 5%); cytopenias
SAFFRON-Lung02 [34]	BL-B01D1 (EGFR/HER3 bispecific ADC)	I	EGFR-mutant NSCLC, TKI-resistant 3rd-gen (*n* = 40)	52.5%	mPFS 11.1 mo	Grade ≥3 TRAEs 67%; leukopenia, anemia

T-DXd: trastuzumab deruxtecan; Dato-DXd: datopotamab deruxtecan; ILD: interstitial lung disease; TROP2: trophoblast cell surface antigen 2; c-MET: mesenchymal–epithelial transition receptor. HER3-DXd: patritumab deruxtecan; TRAEs: treatment-related adverse events; EGFR-mut: EGFR-mutant.

**Table 5:** Bispecific Antibodies vs. Antibody–Drug Conjugates (ADCs): Mechanistic and Clinical Comparison.

Feature	Bispecific Antibodies (bsAbs)	Antibody–Drug Conjugates (ADCs)
Structure	Two antigen-binding arms ± Fc region; no cytotoxic payload [43,44]	Monoclonal antibody + chemical linker + cytotoxic payload (e.g., DXd, MMAE) [36]
Mechanism of action	Dual receptor blockade + immune cell recruitment (ADCC, ADCP); no direct cytotoxic payload delivery [15,41]	Targeted intracellular delivery of cytotoxin; bystander effect via membrane-permeable payloads [36]
Immune effects	Fc-dependent ADCC/ADCP; T-cell/NK-cell redirection (if CD3/CD16 arm) [41]	Limited direct immune effector recruitment compared with Fc-active bsAbs [36]
Key toxicities	IRRs, VTE, dermatologic AEs, hematologic toxicities (with chemo combo) [13,47]	ILD (payload-dependent), cytopenias, nausea, peripheral neuropathy (vedotin-based) [15,48,49,50]
Resistance mechanisms	Antigen escape, Fc receptor polymorphisms, compensatory RTK upregulation, downstream MAPK reactivation [15,41]	Antigen loss, impaired internalization, lysosomal dysfunction, drug efflux (MDR1/ABCB1) [36]
Approved NSCLC agents	Amivantamab (EGFR/MET); zenocutuzumab (HER2/HER3, NRG1 fusion) [15,42]	T-DXd (HER2-mut); telisotuzumab vedotin (c-MET high); Dato-DXd (EGFR-mut post-TKI) [15,48,49,50]
Combination strategy	bsAb + TKI (MARIPOSA); bsAb + chemo (PAPILLON); bsAb + IO: emerging [13,16,51]	ADC + IO; ADC + TKI; sequential ADC strategies; ADC + ADC emerging [36]
Novel hybrid class	Bispecific ADC (BL-B01D1: EGFR/HER3 bsAb + DXd payload) merging both modalities [34]	

ADCC: antibody-dependent cell cytotoxicity; ADCP: antibody-dependent cellular phagocytosis; IRR: infusion-related reaction; VTE: venous thromboembolism; ILD: interstitial lung disease; MDR1/ABCB1: multidrug resistance protein; IO: immunotherapy/immune checkpoint inhibitor; MMAE: monomethyl auristatin E.

**Table 6:** Bispecific Antibodies vs. TKIs: Comprehensive Clinical Comparison.

Domain	Bispecific Antibodies (bsAbs)	TKI Monotherapy
Mechanism	Dual receptor blockade (e.g., EGFR + MET); immune effector recruitment (ADCC, ADCP, trogocytosis) [15,41]	ATP-competitive inhibition of mutant kinase active site; blocks downstream RAS-MAPK and PI3K-AKT signaling [4,32]
Administration/PK	IV infusion q1–2 weeks; long half-life (~14 days for amivantamab); slower pharmacodynamic onset [15]	Oral daily dosing; rapid systemic absorption and biochemical response; short half-life [3]
CNS penetration	Theoretically limited by antibody size (~150 kDa), but comparable intracranial clinical response in trials [13,47]	Brain-penetrant 3rd-gen TKIs (osimertinib, lorlatinib) achieve high CNS exposure [3,6]
Resistance landscape	Less vulnerable to kinase-domain mutations; can overcome MET-driven TKI resistance; susceptible to antigen loss/downregulation [15,41]	Resistance via secondary EGFR mutations (T790M, C797S), MET/HER2 amplification, bypass activation, EMT, SCLC transformation [32,33,35]
Toxicity profile	IRRs (63%), VTE (37%), paronychia, dermatitis, edema; unique immune-mediated AEs [13,47]	Diarrhea, rash, hepatotoxicity; class-specific QTc prolongation or ILD with some agents [3]
Tumor heterogeneity	Can simultaneously address clones expressing multiple oncogene combinations [34,41]	Single-target; clonal heterogeneity can drive rapid resistance emergence [4,32]
Combination potential	Synergistic with TKIs (MARIPOSA), chemotherapy (PAPILLON, MARIPOSA-2); IO partnerships emerging [13,15,47]	Combine with anti-angiogenics, chemotherapy, CDK4/6 inhibitors; emerging TKI–TKI doublets [3]
Current approved role	Post-TKI resistance; EGFR Ex20ins (approved); 1st-line EGFR-mutant + lazertinib (MARIPOSA); NRG1 fusion (zenocutuzumab) [13,16,42]	1st-line SOC for EGFR, ALK, ROS1, RET, KRAS G12C, METex14, BRAF V600E, NTRK mutations [3,6]
Cost/accessibility	High; IV infusion requires clinic attendance; access challenges in resource-limited settings [15]	Oral; generally accessible; generics emerging for older TKI generations [3]

ADCC: antibody-dependent cell cytotoxicity; ADCP: antibody-dependent cellular phagocytosis; SCLC: small cell lung cancer transformation; EMT: epithelial-to-mesenchymal transition; VTE: venous thromboembolism; IRR: infusion-related reaction; SOC: standard of care; IV: intravenous. References embedded in cells.

**Table 7:** Analysis of FLAURA, FLAURA2 and MARIPOSA.

Parameter	FLAURA [7]	FLAURA2 [8]	MARIPOSA [13,14]
Sponsor/Setting	Phase III; AstraZeneca; global	Phase III; AstraZeneca; global	Phase III; Janssen/J&J; global
Population	EGFR Exon 19 deletion/L858R, 1st-line, unselected (*n* = 556)	EGFR Exon 19 deletion/L858R, 1st-line, unselected (*n* = 557)	EGFR Exon 19 deletion/L858R, 1st-line, unselected (*n* = 1074)
Experimental arm	Osimertinib 80 mg QD	Osimertinib 80 mg QD + carboplatin/pemetrexed (4 cycles) -> osi + pem maintenance	Amivantamab IV + lazertinib 240 mg QD
Control arm	Gefitinib or erlotinib (physician choice)	Osimertinib 80 mg QD	Osimertinib 80 mg QD
Primary endpoint	PFS (investigator-assessed)	PFS (investigator-assessed)	PFS (blinded independent central review)
Median PFS—Experimental	18.9 months	25.5 months	23.7 months
Median PFS—Control	10.2 months	16.7 months	16.6 months
HR (PFS); *p*-value	HR 0.46; *p* < 0.001	HR 0.62; *p* < 0.001	HR 0.70; *p* < 0.001
OS—Experimental	38.6 months	Not yet mature	NR (ongoing)
OS—Control	31.8 months	Not yet mature	NR
OS HR; *p*-value	HR 0.80; *p* = 0.046 [52]	NR (interim)	HR 0.75; *p* = 0.005 [14]
Intracranial PFS/iORR	Strong CNS activity (osimertinib historically considered highly CNS-active)	Strong CNS activity; pronounced benefit in patients with baseline brain metastases	Comparable iORR (~77% both arms); superior 36-mo intracranial PFS for amivantamab–lazertinib (36–38% vs. 18%; HR 0.69–0.79) [13]
Grade ≥3 AEs (exp. arm)	~35%	~64% (chemo phase)	~75%
Discontinuation for AE	~7%	~12%	~16% (amivantamab)
Key toxicities	Rash, diarrhea, paronychia, stomatitis	Myelosuppression, nausea, fatigue, alopecia	IRR 63%, VTE 37%, paronychia, rash, peripheral edema
Mechanism of benefit	Single-target TKI kinase inhibition	TKI kinase inhibition + cytotoxic chemo	Dual receptor (EGFR + MET) blockade + ADCC/ADCP immune engagement + TKI kinase inhibition
Resistance implications	Fails via C797S, MET amp, bypass	Delays bypass via chemotherapy elimination of resistant clones	Addresses MET bypass at baseline; novel resistance via antigen escape
Key clinical implication	Standard 1st-line EGFR SOC (3rd gen TKI)	First-line intensification with chemo for high-risk patients	First-line biological dual-targeting for EGFR classic mutations; FDA approved 2024

HR: hazard ratio; OS: overall survival; PFS: progression-free survival; iORR: intracranial objective response rate; VTE: venous thromboembolism; IRR: infusion-related reaction; carbo/pem: carboplatin/pemetrexed; NR: not reached.

## Data Availability

No new data were created or analyzed in this study. Data sharing is not applicable to this article.

## Abbreviations

ADC: antibody–drug conjugate; ADCC: antibody-dependent cellular cytotoxicity; ADCP: antibody-dependent cellular phagocytosis; ALK: anaplastic lymphoma kinase; ATP: adenosine triphosphate; BRAF: v-Raf murine sarcoma viral oncogene homologue B; bsAb: bispecific antibody; CDC: complement-dependent cytotoxicity; CNS: central nervous system; CRS: cytokine release syndrome; ctDNA: circulating tumor DNA; DART: dual-affinity re-targeting; DOR: duration of response; EGFR: epidermal growth factor receptor; EMT: epithelial-to-mesenchymal transition; FDA: Food and Drug Administration; HER2/3: human epidermal growth factor receptor 2/3; HR: hazard ratio; IHC: immunohistochemistry; ILD: interstitial lung disease; IO: immunotherapy/immune checkpoint inhibitor; IRR: infusion-related reaction; KEAP1: Kelch-like ECH-associated protein 1; KRAS: Kirsten rat sarcoma viral proto-oncogene; MET: mesenchymal–epithelial transition proto-oncogene; mDOR: median duration of response; mOS: median overall survival; mPFS: median progression-free survival; NSCLC: non-small-cell lung cancer; NRG1: neuregulin-1; NTRK: neurotrophic tyrosine receptor kinase; ORR: objective response rate; PFS: progression-free survival; PI3K: phosphoinositide 3-kinase; RAS-MAPK: rat sarcoma–mitogen-activated protein kinase; RET: rearranged during transfection proto-oncogene; ROS1: c-ros oncogene 1; RTK: receptor tyrosine kinase; SCLC: small cell lung cancer; SOC: standard of care; STK11: serine/threonine kinase 11; TKI: tyrosine kinase inhibitor; TROP2: trophoblast cell surface antigen 2; VTE: venous thromboembolism.
